# Supplementary material for: Anatomy of the energetic driving force for charge generation in organic solar cells
Source: Nat Commun. 2019 Jun 7;10:2520. doi: 10.1038/s41467-019-10434-3 (PMC6555791; doi:10.1038/s41467-019-10434-3)
Supplement: Supplementary file 1 — Supplementary Information [file 41467_2019_10434_MOESM1_ESM.doc]

**Supplementary information for**

**Anatomy of the Energetic Driving Force for Charge Generation in Organic Solar Cells**

*Kyohei Nakano*^1^*, Yujiao Chen*^1^*,* *Bo Xiao*^2^*, Weining Han*^3^*, Jianming Huang*^1^*,* Hiroyuki Yoshida^3,4^, Erjun Zhou^2,*^ *and Keisuke Tajima*^1,*^

^1^RIKEN Center for Emergent Matter Science (CEMS), 2-1 Hirosawa, Wako, Saitama 351-0198, Japan

^2^CAS Key Laboratory of Nanosystem and Hierarchical Fabrication, CAS Center for Excellence in Nanoscience, National Center for Nanoscience and Technology, Beijing 100190, P. R. China

^3^Graduate School of Engineering, Chiba University, 1-33 Yayoi-cho, Inage-ku, Chiba-shi, Chiba 263-8522, Japan

^4^Molecular Chirality Research Center, Chiba University, 1-33 Yayoi-cho, Inage-ku, Chiba-shi, Chiba 263-8522, Japan

^*^ E-mail: zhouej@nanoctr.cn

^*^ E-mail: keisuke.tajima@riken.jp

**Supplementary Figures**

**Supplementary Figure 1. | UPS spectra of donor polymers.** **a,** Secondary electron cutoff region, **b**, HOMO region. Cutoff energy was evaluated using the intersection of the fitting line. We fitted a single Gaussian to the HOMO band. The HOMO edge was systematically evaluated as Gaussian peak + 2σ, where σ is the width of the Gaussian. The HOMO edge positions evaluated by this Gaussian fit agreed well with the onset of the UPS signal.

**Supplementary Figure 2. | Low-energy inverse photoemission spectroscopy spectra of BTAs**. **a**, BTA1, **b**, BTA2, **c**, BTA3. Left panels show spectra measured with different detector photon energies. Right panels show the onset of the LUMO derived peak in the electron kinetic energy plotted against the detected photon energy. Electron affinity is evaluated using the intersection of the linear fitting line with these three measured points.

**Supplementary Figure 3. |** Schematics of the energy levels of the materials with respect to the vacuum level.

**Supplementary Figure 4. |** **Normalized absorption and photoluminescence (PL) in films.** **a**, P3HT, **b**, PTB7, **c**, PDCBT, **d**, J61, **e**, BTA1, **f**, BTA2, **g**, BTA3 **h**, PCBM. Arrows indicate the absorption and emission cross point, providing *E*_g_^opt^. The excitation wavelength was 590 nm for PTB7, 300 nm for PCBM, and 540 nm for the other films. PCBM did not show a sharp edge for light absorption, making it difficult to evaluate *E*_g_^opt^ directly. Instead, we calculated *E*_g_^opt^ as 1.71 eV (725 nm) by using reported values of the binding energy of the PCBM singlet exciton (0.39 eV)^1^ and the HOMO-LUMO gap of PCBM (2.10 eV)^2^.

**Supplementary Figure 5. |** **Current density-voltage characteristics under AM1.5 100 mW cm^−2^ simulated sunlight irradiation.** **a**, P3HT, **b**, PTB7, **c**, PDCBT, **d**, J61 with four acceptors.

**Supplementary Figure 6. |** **External quantum efficiency of 16 PHJ devices.** **a**, P3HT, **b**, PTB7, **c**, PDCBT, **d**, J61 with four acceptors.

**Supplementary Figure 7. | *V*_OC_ plotted against temperature**. **a**, P3HT, **b**, PTB7, **c**, PDCBT, **d**, J61 with four acceptors. The intersections at 0 K determined by the linear extrapolation of *V*_OC_ were used as the ­*E*_CT_ values. During the measurements, the temperatures of the sample stage (bottom side) and sample surface (top side) were monitored with thermocouples to minimize the error in the sample temperature.

**Supplementary Figure 8. | Spectroscopic measurements for determining the absorption and emission of CT states. a,** P3HT-BTA1, **b,** P3HT-BTA2, **c,** P3HT-BTA3, **d,** P3HT-PCBM, **e,** PTB7-BTA3, **f,** PTB7-PCBM, **g,** PDCBT-BTA3, **h,** PDCBT-PCBM, **i,** J61-BTA3, **j,** J61-PCBM. **(Left panels) Normalized reduced electroluminescence spectra of PHJ devices and photoluminescence spectra of pristine donor and acceptor films.** The applied voltage and resulting current density during the EL measurement are shown in the legends. The orange lines are the gaussian fitting to the EL spectrum. **(Right panels) Reduced EQE and EL spectra of the PHJ devices.** The black solid lines are the gaussian fitting to these spectra. The ­*E*_CT_ was determined as the energy at the cross point of the two gaussians.

**Supplementary Figure 9. | ­*E*_CT_ determined by EQE/EL measurements plotted against *E*_CT_ determined by *qV*_OC_-*T* plots.**

**Supplementary Figure 10. | Light intensity dependence of *J*_SC_ and collected charge**. **a**, BTA1/J61, **b**, BTA3/J61, **c**, BTA3/P3HT. Left panels show the light intensity dependence of *J*_SC_ at different temperatures. Right panels show the amount of collected charges during transient photocurrent (TPC) measurements normalized using those obtained in the dark. In the TPC measurements, the solar cells were irradiated with steady-state LED light and under short-circuit conditions, so that constant current flowed through the cell. Pulsed laser (wavelength: 532 nm, pulse duration: 0.4 ns) irradiation of the cell generated additional transient charge. The collection of this additional charge was recorded by an oscilloscope as a transient current, enabling us to estimate the amount of collected charges by the integral of the current over time. If the non-geminate recombination has a large impact on the charge collection process under short-circuit conditions, the amount of collected charge depends on the steady-state charge density (light intensity). The amount of collected charges was insensitive to the bias that the light intensity showed in the right panels, which indicated that non-geminate recombination had a negligible effect under short-circuit condition.

**Supplementary Figure 11. | Measured and calculated spectra used for reproducing the EQE spectra.** **a,** P3HT-BTA1, **b,** P3HT-BTA2, **c,** P3HT-BTA3, **d,** P3HT-PCBM, **e,** PTB7-BTA1, **f,** PTB7-BTA2, **g,** PTB7-BTA3, **h,** PTB7-PCBM, **i,** PDCBT-BTA1, **j,** PDCBT-BTA2, **k,** PDCBT-BTA3, **l,** PDCBT-PCBM, **m,** J61-BTA1, **n,** J61-BTA2, **o,** J61-BTA3, **p,** J61-PCBM. **(Left panels) Measured and simulated 1−reflectance−transmittance (1−R−T) spectra**. The reflectance and transmittance of PHJs were obtained using an integrating sphere. The experimentally measured and simulated absorptance spectra matched well in all PHJs. **(Center panels) Simulated light absorptance of the donor and acceptor materials of the minimum and maximum exciton collection length (ECL).** The assumed ECLs were 13–18 nm for PCBM, and 7–12 nm for other materials. **(Right panels) Measured and reproduced external quantum efficiency with using the minimum ECL for the donor and acceptor.** The sum of donor and acceptor contributions for the charge generation reproduced the measured EQE spectra well with the exceptions of **e** and **f** (see Supplementary Note 3).

**Supplementary Figure 12. | Charge generation efficiency dependence on *E*_g_^opt^ – *E*_CT_ and *E*_CT_ – *E*_CS_**. Charge generation efficiency plotted against **a,** *E*_g_^opt^ – *E*_CT_, **b,** *E*_CT_ – *E*_CS_. *E*_CT_ values of 10 PHJs were determined by the EQE/EL technique (colored symbols), and by temperature for the other PHJs (black symbols).

**Supplementary Figure 13. | Charge generation efficiency dependence on *E*_g_^opt^ – *E*_CS_ and *E*_g_^opt^ − *E*_CT_**. Charge generation efficiency plotted against **a,** *E*_g_^opt^ – *E*_CS_, **b,** *E*_g_^opt^ − *E*_CT_. The smaller *E*_g_^opt^ value of the donor and the acceptor was used based on the assumption that the excited energy was transferred completely to the material with the lower *E*_g_^opt^.

**Supplementary Figure 14. | Fill factor and energy difference**. Fill factor plotted against **a**, *E*_g_^opt^ – *E*_CT_, **b**, *E*_CT_ – *E*_CS_. A clear correlation only appeared in the *E*_g_^opt^ − ­*E*_CT_ plot, indicating that the decay of the S_1_ state to the CT state has a large impact on FF.

 **Supplementary Figure 15. | Normalized photoluminescence of PHJ cells and pristine films of the donor and acceptor used for the cells**. **a**, BTA3/J61, **b**, PCBM/J61. Excitation wavelength was 500 and 540 nm, respectively. Pristine films were prepared on a fused silica substrate.

 **Supplementary Figure 16. | (Left panels) Normalized photoluminescence of PHJ cell under applied reverse bias. (Right panels) Comparison of photoluminescence of a device with those of the pristine films of the donor and acceptor**. **a**, BTA2/J61, **b**, PCBM/PTB7, **c**, BTA2/P3HT. The excitation wavelengths were 500, 590, and 500 nm for the BTA2/J61 PHJ, PCBM/PTB7, and BTA2/P3HT PHJs, respectively. Pristine films were prepared on a fused silica substrate. BTA2/J61, which has a large electric field dependence of *η*_gen_ (*η*_gen_^A^: 45 %; *η*_gen_^D^: 56 %) exhibited bias dependence of the emission from the S­_1_ state of BTA2, whereas the emissions from the S_1_ states of PTB7 and P3HT in the PCBM/PTB7 and BTA2/P3HT PHJs with small electric field dependence of *η*_gen_^D^ (24% and 10%, respectively) were independent of the applied field.

**Supplementary Figure 17. | Schematic of the energy levels near the D/A interface (left) and when the donor near the D/A interface has a disordered layer (right).** If only the donor phase has a disordered layer, *E*_g_^opt^ of the layer would be increased and ­*E*_CT_ would be increased because of the downward shift of *E*_HOMO_^D^. For the charge generation process at the interfaces of the disordered donor and the acceptor, the energetic driving force (i.e., *E*_g_^opt^ – *E*_CT_) must be calculated by using *E*_g_^opt^ of the disordered layer. However, this broadened, blue-shifted light absorption of the thin layer may overlap with the light absorption of the bulk of the donor. Moreover, the absorption of the disordered layer is expected to be much smaller than that of the bulk because it is a thin layer at the D/A interface. Therefore, the light absorption of the disordered layer at the interface was not detected in the absorption spectra. Consequently, the *E*_g_^opt^ values for the pristine films or the bulk of the donor are usually used for estimating *E*_g_^opt^ – ­*E*_CT_, but this could lead to the underestimation of *E*_g_^opt^ – *E*_CT_ in BHJs.

**Supplementary Tables**

**Supplementary Table 1. | Summary of *E*_CS_, ­*E*_CT,_ and reorganization energy (***λ***) determined by *qV*_OC_-*T* plots and EQE/EL measurements**.

| Acceptor | Donor | *E*_CS_ (eV) | *E*_CT_ by  *V*_OC_-*T* (eV) | *­E*_CT_ by  EQE/EL (eV) | Goodness of gaussian fit | λ (eV) |
| --- | --- | --- | --- | --- | --- | --- |
| BTA1 | P3HT | 1.80 | 1.51 | 1.74 | Moderate | 0.26 |
| BTA2 | P3HT | 2.02 | 1.65 | 1.73 | Moderate | 0.27 |
| BTA3 | P3HT | 1.53 | 1.15 | 1.69 | Moderate | 0.31 |
| PCBM | P3HT | 1.57 | 0.94 | 1.22 | Good | 0.33 |
| BTA1 | PTB7 | 1.89 | 1.80 | - | Bad | - |
| BTA2 | PTB7 | 2.11 | 1.81 | - | Bad | - |
| BTA3 | PTB7 | 1.62 | 1.61 | 1.61 | Moderate | 0.22 |
| PCBM | PTB7 | 1.66 | 1.40 | 1.60 | Good | 0.26 |
| BTA1 | PDCBT | 1.94 | 1.78 | - | Bad | - |
| BTA2 | PDCBT | 2.16 | 1.98 | - | Bad | - |
| BTA3 | PDCBT | 1.67 | 1.61 | 1.72 | Moderate | 0.28 |
| PCBM | PDCBT | 1.71 | 1.40 | 1.35 | Good | 0.29 |
| BTA1 | J61 | 2.08 | 1.81 | - | Bad | - |
| BTA2 | J61 | 2.30 | 1.91 | - | Bad | - |
| BTA3 | J61 | 1.81 | 1.58 | 1.68 | Moderate | 0.35 |
| PCBM | J61 | 1.85 | 1.33 | 1.41 | Good | 0.28 |

**Supplementary Table 2. | Summary of spin-coating conditions, annealing conditions, and film thicknesses of the donor and acceptor film**s**.** Annealing was conducted in a glovebox filled with dry N_2_. Film thickness was measured by X-ray reflectivity, using films prepared on a Si substrate. CB: chlorobenzene; CF: chloroform; EI: ethyl iodide.

|  | Solvent | Concentration (mg mL^−1^) | Rotation speed (rpm) | Annealing  (°C, min) | Thickness (nm) |
| --- | --- | --- | --- | --- | --- |
| P3HT | CB | 10.0 | 1000 | - | 48 |
| PTB7 | CB | 8.0 | 1000 | - | 43 |
| PDCBT | CF:EI, 1:1 | 5.0 | 1000 | - | 47 |
| J61 | CF | 4.0 | 1000 | 100, 10 | 44 |
| BTA1 | CF | 3.0 | 1000 | 100, 10 | 27 |
| BTA2 | CF | 3.0 | 1000 | 100, 10 | 26 |
| BTA3 | CF | 3.0 | 1000 | 100, 10 | 28 |
| PCBM | CB | 10.0 | 600 | 150, 5 | 35 |

**Supplementary Table 3. | Summary of short-circuit current (*J*_SC_), open-circuit voltage (*V*_OC_), fill factor (FF), and power conversion efficiency (PCE) values**. Numbers in parentheses are standard deviations calculated from at least six devices.

| Acceptor | Donor | *J*_SC_ (mA cm^−2^) | *V*_OC_ (V) | FF | PCE (%) |
| --- | --- | --- | --- | --- | --- |
| BTA1 | P3HT | −0.64  (0.11) | 1.05  (0.009) | 0.50  (0.038) | 0.33  (0.048) |
| BTA1 | PTB7 | −0.067 (0.013) | 1.17  (0.064) | 0.18  (0.018) | 0.014  (0.003) |
| BTA1 | PDCBT | −0.27  (0.064) | 1.30  (0.044) | 0.42  (0.071) | 0.152  (0.058) |
| BTA1 | J61 | −0.46  (0.062) | 1.28  (0.032) | 0.35  (0.027) | 0.21  (0.037) |
| BTA2 | P3HT | −0.83  (0.066) | 1.18  (0.059) | 0.48  (0.079) | 0.48  (0.12) |
| BTA2 | PTB7 | −0.026  (6.7E−3) | 1.15  (0.056) | 0.13  (0.012) | 0.0040  (0.0014) |
| BTA2 | PDCBT | −0.13  (7.3E−3) | 1.39  (0.048) | 0.21  (0.005) | 0.037  (0.003) |
| BTA2 | J61 | −0.23  (0.017) | 1.33  (0.027) | 0.21  (0.004) | 0.061  (0.006) |
| BTA3 | P3HT | −1.9  (0.076) | 0.84  (0.015) | 0.58  (0.022) | 0.92  (0.074) |
| BTA3 | PTB7 | −1.6  (0.027) | 1.14  (0.005) | 0.36  (0.025) | 0.67  (0.05) |
| BTA3 | PDCBT | −0.68  (0.0154) | 1.10  (0.006) | 0.41  (0.005) | 0.31  (0.007) |
| BTA3 | J61 | −1.4  (0.051) | 1.12  (0.005) | 0.33  (0.031) | 0.52  (0.038) |
| PCBM | P3HT | −1.2  (0.073) | 0.48  (0.04) | 0.64  (0.024) | 0.37  (0.014) |
| PCBM | PTB7 | −2.1  (0.14) | 0.82  (0.007) | 0.56  (0.051) | 0.97  (0.131) |
| PCBM | PDCBT | −1.6  (0.12) | 0.77  (0.054) | 0.65  (0.018) | 0.80  (0.13) |
| PCBM | J61 | −2.0  (0.20) | 0.74  (0.025) | 0.63  (0.022) | 0.94  (0.15) |

**Supplementary Notes**

**Supplementary Note 1. | Negligible effect of non-geminate recombination on charge collection in PHJs.**

We measured the light intensity dependence of *J*_SC_ and transient photocurrent (TPC) at various temperatures for three devices with different *E*_g_^opt^ − *E*_CT_ values (Supplementary Figure 10). Regardless of the energy difference, *J*_SC_ scaled with the light intensity with a slope of unity^3–5^. Moreover, we found no change in the amount of collected charges during TPC measurements, regardless of the light intensity. These trends persisted even at lower temperatures (~210 K), where the charge mobilities decrease and the effects of the non-geminate recombination are greater. Based on these results, we concluded that the non-geminate recombination in the PHJ cells is negligible under short-circuit conditions and we treated the charge collection efficiency (*η*_col_) at short-circuit as unity. This assumption is not necessarily true for BHJs and can be characteristic of PHJs because the electrons and holes are spatially separated in the thin acceptor and the donor layers, respectively.

**Supplementary Note 2. | Simulation of light absorption profile in PHJ**

We calculated light absorption profiles in the multilayered films by using the optical transfer matrix formalism following a reported procedure^6,7^. The optical constants of the organic materials, MoO*_x_*, ITO, and glass were obtained by spectroscopic ellipsometry. The optical constant of Ag was taken from a materials database. The PEIE buffer layer was ignored in the model because of its thinness and negligible light absorption. The experimentally measured absorptance spectra with an integrating sphere and simulated spectra matched well for all PHJs (Supplementary Figure 11, left panel). The absorptance spectra calculated only for the organic layers are also shown. The large parasitic absorptions of the ITO/glass substrate and the Ag electrode in the OSC spectra in the 300–400 and 700–900 nm regions are visible. These analyses give the full energy dissipation profiles, *η*_abs_(*λ*, *z*), which indicate how much light at each wavelength is absorbed at each position in the multilayered films.

**Supplementary Note 3. | Reproducing EQE spectra using calculated light absorption.**

The experimentally measured EQE spectra were fitted by the least-squares method with the absorption spectra of the donor and the acceptor layers within the minimum and the maximum ECLs and with the charge generation efficiency as fitting parameters. The fitting results with the minimum ECL are shown in Supplementary Figure 11, right panel. EQE spectra calculated with the ECLs and *η*_gen_ matched well with the experimentally measured EQE spectra of most of the systems with a few exceptions. In systems with very low *E*_g_^opt^ − *E*_CT_ (BTA1/PTB7 and BTA2/PTB7), the measured EQE spectra had a strong absorption band around 400 nm that could not be assigned as the absorption of the S_1_ transition of the donor or the acceptor. The peak positions matched the absorption of the S_2_ (or higher) electronic states of BTA1 and BTA2. Generally, the direct charge generation from S_2_ states are weak compared with that from S_1_ states because the S_2_ absorption is weak and the electronic relaxation from S_2_ to S_1_ can compete. However, when the charge generation efficiency from S_1_ states was low owing to an insufficient *E*_g_^opt^ − *E*_CT_ value, the direct charge generation from higher electronic states could compete because the difference between the energy of the S_2_ state and *E*_CT_ could still be enough for the charge generation. We did not include the S_2_ contribution in the EQE calculations because our primary interest was in the relationship between *η*_gen_ and the energy of the lowest excited state.

**Supplementary Note 4. | Effect of energy transfer between the donor and acceptor**

In the main text, we used different values of *E*_g_^opt^ for the donors and acceptors to calculate *E*_g_^opt^ − *E*_CT_. This implicitly assumes that the excited state stays on the same side of the PHJs and the energy transfer between the donor and the acceptor is negligible. However, energy transfer was observed in some of the non-fullerene acceptor/polymer systems. The exact estimation of the efficiency of this energy transfer in the PHJ devices was difficult. Therefore, we assumed the opposite extreme case, that the excited energy was completely transferred to the material with the lower *E*_g_^opt^ and plotted *η*_gen_ against the smaller *E*_g_^opt^ – *E*_CT_ of the donor and the acceptor (Supplementary Figure 13). Even with this assumption, the clear correlation between *η*_gen_ and *E*_g_^opt^ − ­*E*_CT_ remained unchanged.

**Supplementary Note 5. | Gaussian plot in Fig. 4b**

In Fig. 4b, we plot a Gaussian to see how our data are explained by Marcus theory. We used the equation,

$$\eta_{\mathrm{gen}}\propto\exp\left[ -\frac{q\left\{ 0.3-\left( E_{g}^{\mathrm{opt}}-E_{\mathrm{CT}} \right) \right\}^{2}}{4\times0.3\times\kappa_{B}T} \right]$$

, where q is elementary charge, *κ*_B_ is Boltzmann constant, *T* is temperature, and 0.3 is reorganization energy. The pre-factor of the exponential function was 0.8. This plot implicitly assumed that the charge generation efficiency was proportional to the charge transfer rate predicted in Marcus theory.

**Supplementary References**

1. Gerhard, M. *et al.* Field-induced exciton dissociation in PTB7-based organic solar cells. *Phys. Rev. B* **95**, 195301 (2017).

2. Zhong, Y. *et al.* Crystallization-Induced Energy Level Change of [6,6]-Phenyl-C61-Butyric Acid Methyl Ester (PCBM) Film: Impact of Electronic Polarization Energy. *J. Phys. Chem. C* **119**, 23–28 (2015).

3. Gebeyehu, D. *et al.* Highly efficient p–i–n type organic photovoltaic devices. *Thin Solid Films* **451–452**, 29–32 (2004).

4. Riedel, I. *et al.* Polymer solar cells with novel fullerene-based acceptor. *Thin Solid Films* **451–452**, 43–47 (2004).

5. Schilinsky, P., Waldauf, C. & Brabec, C. J. Recombination and loss analysis in polythiophene based bulk heterojunction photodetectors. *Appl. Phys. Lett.* **81**, 3885–3887 (2002).

6. Persson, N.-K. & Inganäs, O. Simulations of optical processes in organic photovoltaic devices. in 107–138 (CRC Press, 2005).

7. Pettersson, L. A. A., Roman, L. S. & Inganäs, O. Modeling photocurrent action spectra of photovoltaic devices based on organic thin films. *J. Appl. Phys.* **86**, 487–496 (1999).
