# Supplementary material for: Anatomy of the energetic driving force for charge generation in organic solar cells
Source: Nat Commun. 2019 Jun 7;10:2520. doi: 10.1038/s41467-019-10434-3 (PMC6555791; doi:10.1038/s41467-019-10434-3)
Supplement: Supplementary file 3 — Solar Cells Reporting Summary [file 41467_2019_10434_MOESM3_ESM.pdf]

nature research

Corresponding author(s): Keisuke Tajima

Solar Cells Reporting Summary

Nature Research wishes to improve the reproducibility of the work that we publish. This form is intended for publication with all accepted papers reporting the characterization of photovoltaic devices and provides structure for consistency and transparency in reporting. Some list items might not apply to an individual manuscript, but all fields must be completed for clarity.

For further information on Nature Research policies, including our data availability policy, see Authors & Referees.

Experimental design

Please check: are the following details reported in the manuscript?

1. Dimensions

Item	Yes/No	Details
Area of the tested solar cells	☒ Yes ☐ No	Methods
Method used to determine the device area	☒ Yes ☐ No	Methods

2. Current-voltage characterization

Item	Yes/No	Details
Current density-voltage (J-V) plots in both forward and backward direction	☐ Yes ☒ No	We did not test the hysteresis behavior.
Voltage scan conditions<br>*For instance: scan direction, speed, dwell times*	☒ Yes ☐ No	Methods
Test environment<br>*For instance: characterization temperature, in air or in glove box*	☒ Yes ☐ No	Methods
Protocol for preconditioning of the device before its characterization	☐ Yes ☒ No	No special preconditioning was performed.
Stability of the J-V characteristic<br>*Verified with time evolution of the maximum power point or with the photocurrent at maximum power point; see ref. 7 for details.*	☐ Yes ☒ No	We did not test it.

3. Hysteresis or any other unusual behaviour

Item	Yes/No	Details
Description of the unusual behaviour observed during the characterization	☐ Yes ☒ No	We did not test it.
Related experimental data	☐ Yes ☒ No	We did not test it.

4. Efficiency

Item	Yes/No	Details
External quantum efficiency (EQE) or incident photons to current efficiency (IPCE)	☒ Yes ☐ No	Supplementary Figure 6.
A comparison between the integrated response under the standard reference spectrum and the response measure under the simulator	☐ Yes ☒ No	We did not do it.
For tandem solar cells, the bias illumination and bias voltage used for each subcell	☐ Yes ☒ No	No tandem device was used.

5. Calibration

Item	Yes/No	Details
Light source and reference cell or sensor used for the characterization	☒ Yes ☐ No	Methods
Confirmation that the reference cell was calibrated and certified	☒ Yes ☐ No	Methods

Calculation of spectral mismatch between the reference cell and the devices under test

- [ ] Yes
- [x] No

We did not do this.

6. Mask/aperture

Size of the mask/aperture used during testing

- [x] Yes
- [ ] No

Methods

Variation of the measured short-circuit current density with the mask/aperture area

- [ ] Yes
- [x] No

We did not test this.

7. Performance certification

Identity of the independent certification laboratory that confirmed the photovoltaic performance

- [ ] Yes
- [x] No

We did not do this. The Performance is not main topic of our paper.

A copy of any certificate(s)
Provide in Supplementary Information

- [ ] Yes
- [x] No

We did not.

8. Statistics

Number of solar cells tested

- [x] Yes
- [ ] No

Supplementary Table 3

Statistical analysis of the device performance

- [x] Yes
- [ ] No

Average and standard deviation are provided.

9. Long-term stability analysis

Type of analysis, bias conditions and environmental conditions
For instance: illumination type, temperature, atmosphere humidity, encapsulation method, preconditioning temperature

- [ ] Yes
- [x] No

We did not test this.
