# Supplementary material for: Anatomy of the energetic driving force for charge generation in organic solar cells
Source: Nat Commun. 2019 Jun 7;10:2520. doi: 10.1038/s41467-019-10434-3 (PMC6555791; doi:10.1038/s41467-019-10434-3)
Supplement: Supplementary file 4 — Source Data [file 41467_2019_10434_MOESM4_ESM.zip › OpticalSim/Readme.docx]

# OpticalSim

Optical multilayer simulator for bilayer organic photovoltaics.

This code simulates transmittance, absorption and reflection spectra of multilayer thin films on a substrate by using transfer matrix formalism. The details of the optical model are described in the literature [Pettersson et al., J. Appl. Phys. 1999, 86, 487]. The substrate is treated as an incoherent layer considering the multiple reflections and the absorption. Only the normal angle for the light incident is considered. Based on the calculated electric field and the energy dissipation distributions, external quantum efficiency of the bilayer-type organic photovoltaics is calculated based on a simple exciton diffusion model and designated charge separation efficiencies at the interface.

How to use:

1. First, you need to collect the optical constants (n and k) of the target wavelength range for the substrate and each layer by spectral ellipsometry. Save them as .nk files. The file format is tabulated wavelength (nm) with the interval of 1 nm, n and k in each line.
2. Describe the layer structure (stack) in .stk files. Each line represents the properties of the layers. The line starts the layers from the top (i.e. light incident side) to the bottom. The first line must be the transparent substrate that is treated as an incoherent layer. Each line contains tabulated items in the following order.

1. Name of .nk file

2. Thickness (nm)

3. Whether the layer is photoactive (1) or not (0)

4. Charge separation efficiency at the top interface (0-1)

5. Charge separation efficiency at the bottom interface (0-1)

6. Exciton diffusion length (nm)

7. Lifetime of exciton (ns) (currently not in use)

8. Whether the top side is quenching (1) or not (0) (currently not in use)

9. Whether the bottom side is quenching (1) or not (0) (currently not in use)

1. Run CmdMultilayer.m and choose the .stk file to simulate.
2. After the calculation, the following variables contains the results:

Alayer: Array of [wavelength (nm), absorption] for each layer.

Atotal: Array of [wavelength (nm), total absorption] for the film (including the absorption of the substrate).

Rtotal: Array of [wavelength (nm), total reflectance] for the film.

Ttotal: Array of [wavelength (nm), total transmittance] for the film.

Qoutput: Array of [grid position (nm), energy dissipation (a.u.)] for each wavelength. The integration of this number within the grid gives absorption in the corresponding region.

Efield: Array of [grid position (nm), electric field/incident electric field] for each wavelength.

1. EQE_constEDL.m calculate the EQE profile by using the absorption profiles assuming the constant exciton collection length (ECL) from the charge separation interface (i.e. the charge separation efficiency is constant within ECL range and zero outside). Different efficiency can be assumed for each side of the interface (the parameters 4 and 5).

Memo: to calculate the absorption of the substrate

The substrate is treated as an incoherent layer considering the multiple reflections and the absorption. Defining the reflectances at air/substrate and substrate/stack interfaces as *R*_sub_ and *R*_stack_, respectively, and the transmittance of the substrate as *T*_sub_, the total reflectance *R*_total_ and the total transmittance *T*_total_ are:

$$R_{\mathrm{total}}=R_{\mathrm{sub}}+\frac{\left( 1-R_{\mathrm{sub}} \right)^{2}{T_{\mathrm{sub}}}^{2}R_{\mathrm{stack}}}{1-R_{\mathrm{sub}}R_{\mathrm{stack}}{T_{\mathrm{sub}}}^{2}}$$

$$T_{\mathrm{total}}=\frac{\left( 1-R_{\mathrm{sub}} \right)T_{\mathrm{sub}}T_{\mathrm{stack}}}{1-R_{\mathrm{sub}}R_{\mathrm{stack}}{T_{\mathrm{sub}}}^{2}}$$

To calculate the electric field and *Q* with the substrate, the following factor should be taken into account compared to the stack in the air. *n*_g_ is refractive index of the substrate.

$$\frac{1-R_{\mathrm{sub}}}{n_{g}\left( 1-R_{\mathrm{sub}}R_{\mathrm{stack}} \right)}$$
